# Ceria Nanotube Formed by Sacrificed Precursors Template through Oswald Ripening

**DOI:** 10.1371/journal.pone.0132536

**Published:** 2015-07-07

**Authors:** Laixue Pang, Xiaoying Wang, Xinde Tang

**Affiliations:** School of Material Science and Engineering, Shandong Jiaotong University, Jinan, P.R China; Institute for Materials Science, GERMANY

## Abstract

Controllable preparation of ceria nanotube was realized by hydrothermal treatment of Ce(OH)CO_3_ precursors. The gradually changing morphologies and microstructures of cerium oxide were characterized by X-ray powder diffraction, scanning electron microscopy and transmission electron microscopy. A top-down path is illuminated to have an insight to the morphological transformation from nanorod to nanotube by adjusting the reaction time. The growth process is investigated by preparing a series of intermediate morphologies during the shape evolution of CeO_2_nanostructure based on the scanning electron microscopy image observation. On the basis of the time-dependent experimental observation, the possible formation mechanism related to oriented attachment and Oswald ripening was proposed, which might afford some guidance for the synthesis of other inorganic nanotubes.

## Introduction

Cerium oxide (CeO_2_) is a technological important material due to its wide applications such as catalyst, fuel cell, sensor, UV shielding, and luminescence. It is widely known that the photocatalytic, magnetic, electronic, and catalytic properties of CeO_2_ are strongly size/shape dependent at the nanometer scale [1–5]. Recent studies in CeO_2_ system have focused on the development of robust synthetic approaches toward size/shape-controlled nanostructures (wires, rods, tube), and the investigation of their size/shape-dependent properties [6–10]. For example, some groups prepared size-tunable CeO_2_ nanocrystals via various wet chemical approaches (including modified precipitation, alcohothermal treatment, microemulsion, and sonochemical method)and investigated their size-dependent UV absorption behavior in order to clarify the confinement effects in CeO_2_[11–15]. The nanotube of ceria has recently attracted a great deal of attention due to the aesthetic beauty and potential unique physical properties. Zhang *et al* prepared CeO_2_ nanotubes using carbon nanotubes as templates by a liquid deposition method [16]. Boehme *et al* synthesized ceria nanotube with diameter of below 100nm and a wall thickness of around 10nm using electroless deposition based on aqueous solutions at room temperature [17]. Hua *et al* fabricated ceria nanotubes by the ultrasonic assisted successive ionic layer adsorption and reaction method to increased amounts of oxygen vacancies and single electron defects containing Ce^3+^ [18]. Tang *et al* developed an approach for high-yield synthesis of single-crystalline CeO_2_ nanotube with a well-shaped hollow interior through a “casually-modified” approach based on the hydrothermal treatment of Ce(OH)CO_3_ precursors with a alkali solution in an aqueous phase[19]. Chen *et al* prepared ceria nanotubes with significantly smaller diameters through hydrothermal treatment of Ce(OH)CO_3_ with dilute NaOH at a mild temperature (120°C)[20]. Among these reported approaches, hydrothermal synthesis has been most extensively investigated because it is simple and cost effective [21–23]. Hydrothermal reaction under moderate conditions is an effective approach in synthesizing nanotube of inorganic oxide [24–27]. In many cases, alkaline solutions are used in the hydrothermal synthesis in which the shape and size of the nanotube are well-controlled. Despite remarkable progress in CeO_2_nanotubessynthesis, the basic formation mechanism is not fully understood, which may be ascribed to the absence of direct experimental observation of the nanotube formation during the growth process. It may be beneficial not only to further understand the growth process, but also to explore the appropriate growth conditions of the produced nanotubes. Therefore, direct experimental determination of such process is of great scientific significance.

In this study, we use scanning electron microscopy (SEM) to obtain and probe intermediate products of hydrothermal synthesis of CeO_2_nanotube. Just by adjusting the hydrothermal treatment time, the morphology transformation from precursor to nanotube is achieved,and series of condition-dependent experiments have been conducted to understand the characteristics of the crystal growth and hollow tube formation processes involved in this synthesis. Furthermore, a possible crystal growth and hollowing mechanism are proposed based on the detailed experimental results.

## Experimental

### Materials and synthesis procedure

Cerium Nitrate (Ce(NO_3_)_3_·6H_2_O), urea(CO(NH_2_)_2_), which received from Sinopharm Chemical Reagent Co. Ltd, were of analytical grade and used as received without further purification. Deionized water was used as the solvent in all experiments.

Rodlike Ce(OH)CO_3_ precursors were synthesized by reacting cerium nitrate with urea. In a typical synthesis, 4mmol of Ce(NO_3_)_3_·6H_2_O and 24mmol of urea were added to 80mLof water under vigorous magnetic stirring. The clear solution was charged into a 100mL wide-mouthed jar which was closed and kept at 80°C for 24 h. The solution was then air-cooled to room temperature. The obtained powder samples were centrifuged, washed with distilled water, and dried at 60°C in air overnight.

**Fig 1:**
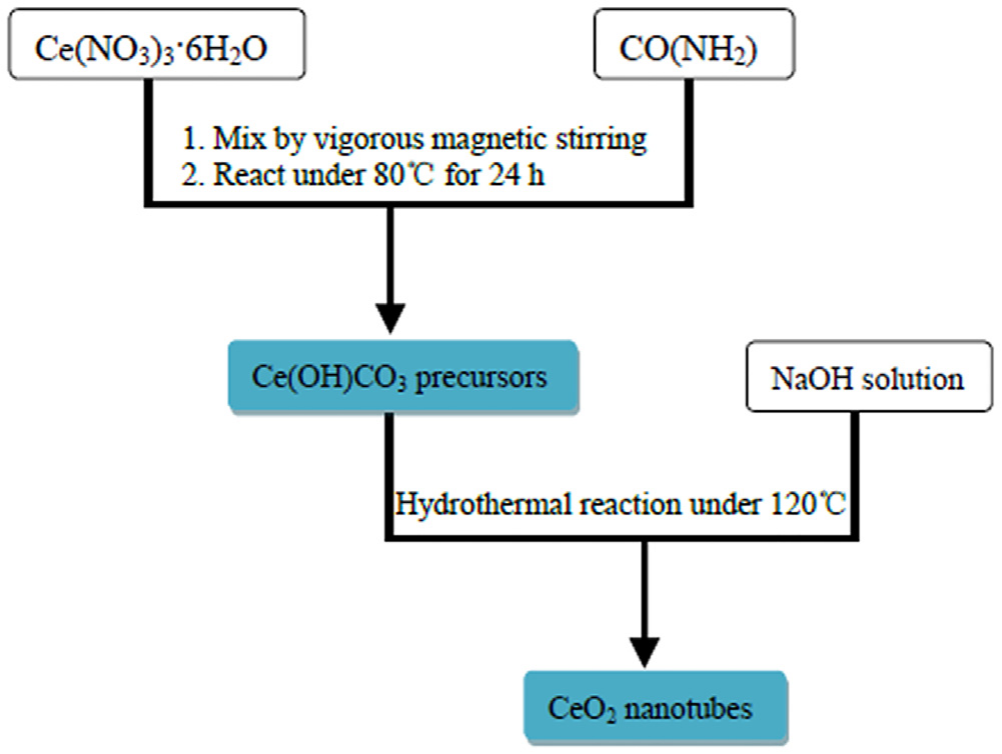
Synthesis strategy flow chart.

The Ce(OH)CO_3_nanorods obtained above were re-dispersed into 20 mL distilled water. Upon adding NaOH solution, the mixture solution was transferred to a Teflon-lined stainless steel autoclave and maintained at 120°C for different duration time (18h, 24h, 48h, 54h, 60h); it was then air-cooled to room temperature. The resulting products were collected, washed several times with absolute ethanol and distilled water, and then dried in a vacuum condition. Flow chart of synthesis strategy for CeO_2_nanotubesis shown as Fig 1.

### Characterization

The obtained samples were characterized by X-ray powder diffraction using a Rigaku D/max-ga X-ray diffractiometer with graphite-monochromatized Cu Kα radiation (λ = 1.54178Å). The morphology and structure of the sample was obtained from transmission electron microscopy (JEM2010 200kV) and field emission scanning electron microscopy (JEOL 6300, 100kV).

## Results and Discussion

**Fig 2:**
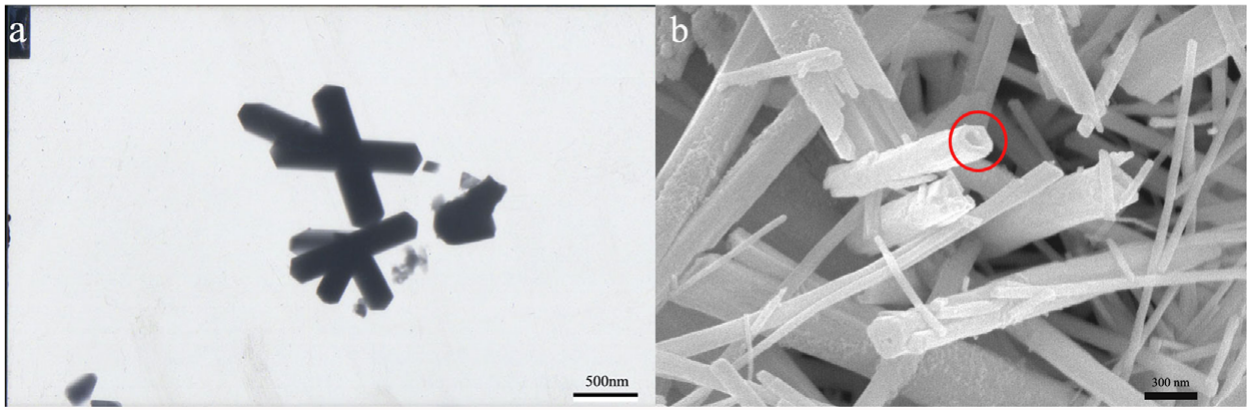
(a)TEM image of as-prepared Ce(OH)CO_3_ precursors and (b) SEM image of CeO_2_ nanotubes.

Representative microscopic images of the as-maderodlike Ce(OH)CO_3_ and as-obtained CeO_2_ nanotubes are as follows. TEM image (Fig 2a) shows a typical morphology of these precursors, which revealing a one-dimension structure under the conditions used. It can be seen that the nanorods have a diameter around 200~300nm, with length typically larger than 1μm. SEM image (Fig 2b) shows the synthesized CeO_2_ nanotube sample, clearly displaying the formation of hollow interiors (red circle), with a well-shaped hollow interior. The diameter of the tube is about 50–100nm.

**Fig 3:**
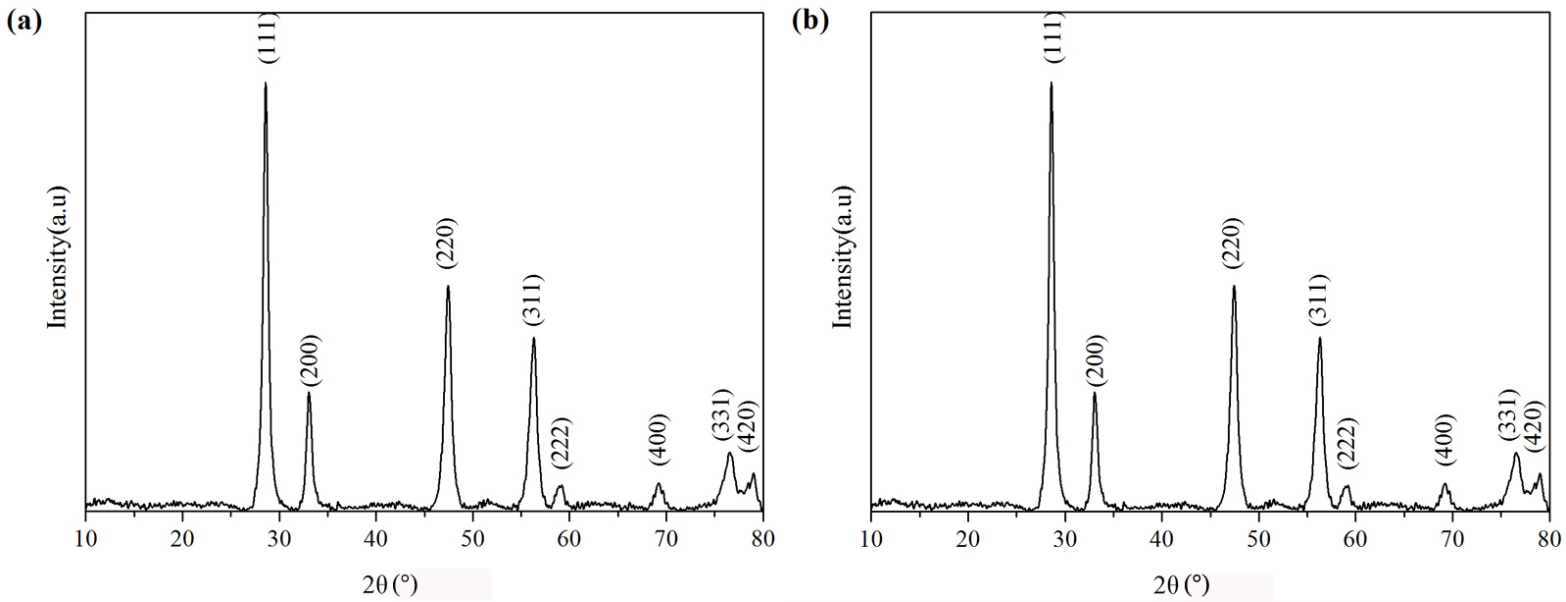
XRD patterns of the as-synthesizde Ce(OH)CO_3_ precursors (a) and CeO_2_ nanotubes (b).

Fig 3 shows a typical XRD pattern of the as-synthesized Ce(OH)CO_3_ and CeO_2_ nanotubes. All peaks in the Fig 3a can be well-indexed to a pure hexagonal phase of Ce(OH)CO_3_ (space group:P $\bar{6}$ 2c) with calculated lattice constants a = 1.252nm and c = 1.000nm, which is in good agreements with the JCPDS file for Ce(OH)CO_3_ (JCPDS 52–0352). No impurity peaks are observed, indicating a high purity of the final products. In Fig 3b all peaks can be indexed as the cubic phase (*Fm* $\bar{3}$ *m*, JCPDS 34–0394) with a lattice constant *a* = 0.5411nm. The strong and sharp diffraction peaks indicate the good crystallization of the sample. No obvious peaks corresponding to cerium nitrate or other cerium oxides were observed in the powder pattern.

**Fig 4:**
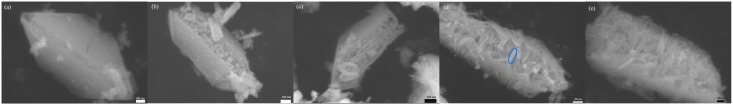
SEM images of the as-prepared CeO_2_ products after different reaction time: (a)6h, (b)12h, (c)18h, (d) 24h, (e) 48h, (f) 54h, (g)60h.

In order to obtain a complete view of the CeO_2_ nanotube formation process and its growth mechanism, the detailed time-dependent evolution of the morphology was evaluated thoroughly by SEM (Fig 4). A clear time-dependent morphology evolution process from the precursors to tubelike shapes can be observed. As shown in Fig 4a, it is obvious that,at the early reaction time, the precursors keep spindle-like morphology. Crystallites growth makes the precursor small crevice. When the reaction time was prolonged to 24h, the crystallites serving as new starting growth sites are growing into cube-shape with different crystal planes due to its anisotropic growth [28]. The loosely packed particles were verified by plenty of intercrystallite spaces observed in these premature cubic structures, as shown in Fig 4c. With the reaction time increasing, the long cylinder-shaped topology is grown, which suggests that the preferred growth of the ceria polyhedra is along the specific direction, similar to that of the CeO_2_nanorods obtained by other methods [29, 30]. The nanocrystals fuse together, forming interfaces among the aggregates, and with time going, those interfaces become lesser, and the nanoparticles merge together and share the same single crystallographic orientation, which leads to the formation of long elongated rod. Adirectionalattachmentgrowth is the major mechanism in this section.

When the reaction time is up to 54h, hollowing takes place and results in the creation of central space indicated by the ruptured nanotube (blue circle in Fig 4d). The hollow shape is formed because cerium tends to move towards the wall of the rod due to the density variation among the rod and then undergo Ostwald ripening process. Due to the difference of surface energy and particles located in the inner space of the cubes and this particles could be dissolved and merged by particles in the outer surface, and meanwhile the solid rod gradually develops into a hollow structures [31–33]. The large nanotubes grow up at the expense of the nanorodwall dissolution, as confirmed by the transparent surface. At last, the precursor is consumed at all; the reaction has run to completion. The perfect hollow structure can be observed as shown in Fig 4e. It has been noted that during the creation of nanotube, the exterior appearance of the precursors did not change appreciably. Therefore, by controlling the hydrothermal time, the hollow interior structure can be effectively monitored, which compared with Kirkendall diffusion mechanism as reported in Ref.[34].

**Fig 5:**
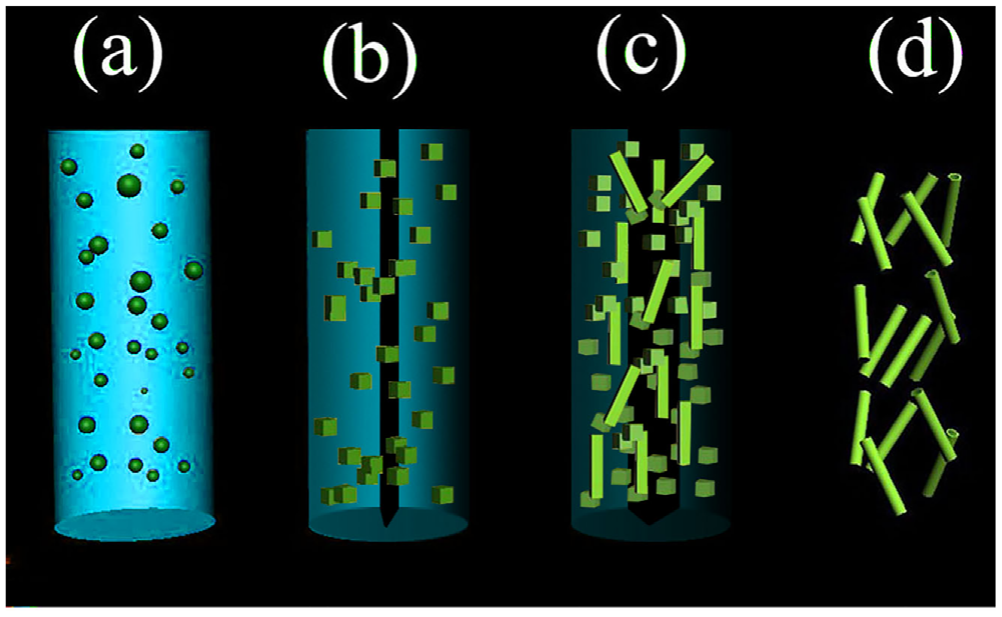
A schematic diagram showing growth mechanism of CeO_2_ nanotubes.

Based on the experimental observations, a possible formation mechanism of CeO_2_ nanotubes is proposed and displayed in Fig 5. At the early stages, the initial nanoparticles are expected to randomly aggregate to reduce the surface energy (Fig 5a). Along with the reaction proceeding, the Ostwald ripening is dominant, and the small, less crystalline particles in a colloidal aggregate dissolved gradually, while larger, better crystallized particles in the same aggregate grew (Fig 5b). Meanwhile, this process involves spontaneous self-organization of adjacent particles so that they share a common crystallographic orientation, followed by the joining of these particles at a planar interface (Fig 5c). At last, the Ostwald ripening is completed with “solid-solution-solid” mass transportation. Crystallites located in the outermost surface ofaggregates are larger and would grow at the expense of smallerones inside, so the solid evacuation occurred. As a result, the CeO_2_ nanotube was formed (Fig 5d). CeO_2_nanostructure with hollow interior space maybe a good CO conversion support owe to its high surface area, which is believed to widely used in catalytic systems.

## Conclusions

In summary, we unveiled the CeO_2_ nanotube shape evolution using the scanning electron microscopy. The morphological evolution can be achieved by adjusting the hydrothermal treatment time. Based on the evidence of electron microscopy images, the morphological evolution mechanism suggested that the nanotube formation might be an Oswald ripening and mass transportation. Considering the convenience of the procedure and the availability of the chemicals used in this ceria nanotube preparation, this route is promising and may be extended to fabricate other metal oxide nanostructures.
